# A machine-learning approach for pancreatic neoplasia classification based on plasma extracellular vesicles

**DOI:** 10.3389/fonc.2025.1540195

**Published:** 2025-04-25

**Authors:** Ioanna Angelioudaki, Angeliki Iosif, Konstadina Kourou, Alexandros-Georgios Tzingounis, Vassiliki Kigka, Androniki-Maria Skreka, Myrto Costopoulos, Nikolaos Memos, Agapi Kataki, Manousos M. Konstadoulakis, Dimitrios I. Fotiadis

**Affiliations:** ^1^ 2^nd^ Department of Surgery, Aretaieion Hospital, Medical School of Athens, National and Kapodistrian University of Athens, Athens, Greece; ^2^ Unit of Medical Technology and Intelligent Information Systems Department of Materials Science and Engineering, University of Ioannina and Biomedical Research Institute, Foundation for Research & Technology - Hellas (FORTH), Ioannina, Greece; ^3^ Flow Cytometry Department, BioAnalytica SA, Athens, Greece; ^4^ 1^st^ Department of Propaedeutic Surgery, “Hippokratio” General Hospital, Medical School of Athens National and Kapodistrian University of Athens, Athens, Greece

**Keywords:** pancreatic cancer, neuroendocrine neoplasms, extracellular vesicles, liquid biopsy, flow cytometry, machine learning

## Abstract

**Introduction:**

Pancreatic cancer (PC) is a lethal disease developing from either exocrine or endocrine cells. Efforts to assist early diagnosis focus on liquid biopsy methods, and especially on the detection of Extracellular Vesicles (EVs) secreted from cancer cells in their microenvironment and accumulated in systemic circulation. Multiple studies explore how EVs size, surface biomarkers or content can determine their unique role and function in the recipient cell’s gene expression, metabolism and behavior affecting cancer development. This study aimed to develop a machine learning-driven (ML) pipeline utilizing clinical variables and EV-based features to predict the presence of pancreatic tumors of different nature (exocrine/endocrine) in patients’ plasma compared to patients with benign lesions or age-matched non-oncological patients.

**Methods:**

All available plasma samples (N=126) and variables were collected prior to surgery. EVs were detected and characterized by flow cytometry-immunostaining. Data including size and a unique set of biomarkers (CD45, CD63 and EphA2) were combined with hematological/biochemical data and processed under two use cases, each formulated as a 3-class classification problem for patient risk stratification. The first use case aimed at classifying patients as with benign lesions or exocrine/endocrine neoplasms. The second use case aimed to distinguish patients with exocrine/endocrine neoplasms from non-oncological patients. Various ML methods were applied, including Logistic Regression, Random Forest, Support Vector Machines, and Extreme Gradient Boosting. Evaluation metrics, as area under the receiver operating characteristic curve (AUC-ROC), were computed, and Shapley values were utilized to determine features with the greatest impact on the discrimination of outcome groups.

**Results:**

Analyses identified hematological and biochemical features, among significant predictors. Models demonstrated substantial accuracy and AUC-ROC values based on plasma EVs subpopulations, which scored over 0.90 in accuracy of the Random Forest and XGBoost algorithms, presenting 0.96 +/- 0.03 accuracy in the first use case and 0.93 +/- 0.04 in the second.

**Discussion:**

By leveraging advanced analytical ML-driven approaches and integrating diverse data types, this study achieved significant accuracy, assisting patient’s risk estimation and supporting the feasibility for early detection of pancreatic cancer. Going beyond currently used biomarkers such as CEA, or CA19.9, EV-based features represent an added value offering increased diagnostic capacity.

## 1 Introduction

Pancreatic cancer (PC) is a lethal disease manifesting as an extremely aggressive neoplasia that develops from either exocrine or endocrine cell populations within the pancreas. The most common form of PC is pancreatic ductal adenocarcinoma (PDAC), accounting for over 90% of cases. The second most common form, pancreatic neuroendocrine neoplasms (pNENs), comprises less than 5% of cases (1). Differentiating between PDAC and pNENs can be challenging especially in atypical patterns, requiring specific imaging techniques and expertise (2). Subtle clinical symptoms and lack of sufficiently accurate screening tests result in delayed diagnosis at the advanced stages of the disease, deterring the option of surgical resection or successful treatment, leading to very poor survival outcomes. Current predictions support that by 2030 PC will be the most fatal type of cancer in the gastrointestinal tract and the second leading cause of cancer-related deaths (3, 4). At the same time, the prevalence of incidental pancreatic cystic neoplasms (pseudocyst, inflammatory cyst, mucinous neoplasm, intraductal papillary mucinous neoplasm) as well as heterogeneous and often unpredictable entities such as pancreatic neuroendocrine neoplasms have increased dramatically during the last decades due to advancements in cross-sectional imaging (5).

While significant research is dedicated to liquid biopsies owing to their potential advantages for cancer diagnosis, prediction, and surveillance, there is an increasing need for detection, validation, assay optimization, and standardization of PC-specific biomarkers. Microparticles as constituents of liquid biopsy, were initially adopted as a term for describing the small membrane-bound vesicles released from cells with a typical diameter ranging from 100nm to 1μm. Later, the term Extracellular vesicles (EVs) came into play which encompasses a broader range of vesicles including exosomes (30-150nm), microvesicles (150-1μm), apoptotic bodies (50–5000 nm), migrasomes (500–3000 nm) and large oncosomes (1000–10 000 nm) (6–8). This shift in terminology reflects an evolving understanding of their complexity and diverse roles in cell communication as well as an emerging need for standardization in their study and application (9). EVs have gained increasing interest because of their primary roles in the development and progression of cancer and in modulating tumor growth and metastasis. These nanosized membrane-bound vesicles, which are secreted by cells (cellular or subcellular origin), carry a unique and diverse array of proteins, DNA, miRNAs, and lipids, either on their surface or within their lumen and they are found elevated in cancer patients’ plasma (10, 11). Emphasis is currently given on how their size, cell of origin, surface biomarkers, or content can determine their unique role and function, with a great impact on the recipient cell’s gene expression, metabolism, and behavior (12). Recent studies have underscored the ability of EVs to modulate the tumor microenvironment, contributing to cancer progression through specific molecular signaling that promotes cell migration, enhances tumor aggressiveness or initiates the formation of a metastatic niche (13, 14).

Up to date, many approaches have been adopted to obtain valuable information on the physical properties and molecular profile of EVs and analyze them on a single-particle level. One approach is by immunostaining and flow cytometry for surface marker confirmation. Common EVs biomarkers include tetraspanins CD9, CD63, and CD81, while CD45 is frequently used as a marker to exclude EVs derived from the hematopoietic system. Furthermore, combinations of other biomarker profiles such as CK-positive, Vim-positive, DAPI-negative, and CD45-negative/CD31-negative in oncosome liquid-biopsy have been comprehensively described through immunofluorescence protocols, correlating their rare-event frequencies to metastatic colorectal cancer patient outcomes (15). Although, several biomarkers have been described enriched in PC-derived EVs, including Ephrins, Glypican-1 (GPC1) and Mucins (MUC1, MUC4, MUC5AC), there is no consensus on a universal lesion-derived specific biomarker (16). Ephrin type-A receptor 2 (EphA2) was identified in 1990, and various *in vitro* studies have described its role as a powerful oncoprotein that is highly overexpressed in cancer cells and is sufficient to confer malignant potential on non-transformed epithelial cells in the surrounding stroma or vasculature. It plays an important role in cell-cell communication in endocrine pancreas, while its overexpression correlates with poor prognosis in patients with PC (17, 18). Recent evidence has shown that EphA2 expressed on the surface of EVs from plasma, can be used to distinguish PDAC patients from healthy subjects (19). Moreover, it was related to the development of chemoresistance, since gemcitabine-sensitive cells become resistant as a result of EphA2 transfer through exosomes from gemcitabine-resistant PC cells (20).

The limitations of traditional diagnostic tools, which often rely on imaging techniques and biomarker detection with limited sensitivity and specificity for early-stage disease, make it difficult to diagnose pancreatic cancer at a very early-stage. These tools struggle to identify subtle molecular signatures or distinguish PC from benign lesions. A potential technique to improve the sensitivity and specificity of PC diagnosis is the targeted analysis of cancer-related EVs. To enhance early diagnosis, EV-based characteristics, such as protein expression, have been used in several studies, occasionally in conjunction with conventional tumor markers or clinical aspects to identify potential biomarkers. While machine learning (ML) methods have been widely adopted for the diagnosis of various other types of cancer, there is notable lack of studies applying these techniques specifically to EV-associated proteins for PC diagnosis. However, advanced statistical methods have been employed to classify PC patients from individuals with benign conditions and healthy controls, with high accuracy (21–24).

In the modern era of Personalized Medicine, the implementation of artificial intelligence (AI) models is slowly growing into standardized practices for tumor detection and classification, which could improve early diagnosis, prognosis, overall survival, and response to therapy predictions. The present study aimed to combine EV features derived from FCM-immunostaining as liquid biopsy method, with novel statistical approaches to distinguish patients with exocrine tumors or endocrine neoplasms from patients with benign lesions or non-oncological patients. The current approach whose innovation lies in its sensitivity and accuracy all the way from features collection to ML algorithms, paves the way towards the adoption of minimally invasive systems with enhanced efficiency in clinical practice.

## 2 Materials and methods

### 2.1 Patient enrollment

Study cohort included 126 plasma samples from patients with exocrine tumors (pancreatic ductal adenocarcinoma n=72, endocrine neoplasms (neuroendocrine tumors and neuroendocrine carcinomas) n=12, benign lesions (Serous Cystadenoma, Pseudocysts, PANIN, IPMN, Mucinous Cystic Pancreatic Neoplasms) n=16, as well as age-matched non-oncological patients n=26. For the purpose of the study, 2ml of blood was collected in EDTA coated tubes (BD vacutainers) from each patient prior to surgical intervention after a typical fasting period of at least 12 h.

### 2.2 Clinical data collection

Demographic and hematological data were obtained from patients’ clinical records. All patients or their legal representatives provided written informed consent for review of their data for research purposes. Part of the plasma samples analyzed were from the “Pancreas” REDCap biobank (blood and tissue samples) approved by the Ethics and Deontology Committees of Aretaieion Hospital (237/10-7-2020) and of Hippokrateio General Hospital of Athens (34/14-7-2020). The current study was conducted in accordance with the Declaration of Helsinki and approved by the Ethics and Deontology Committee of Aretaieion Hospital (488/20-02-2023).

### 2.3 Extracellular vesicles – based features collection

Collected plasma was differentially centrifuged: samples were spun down consecutively at 400g-1200g-10,000g to remove any remaining cells or cell debris. The supernatant was aliquoted and stored frozen at -80°C until use for downstream analysis. EVs were detected and characterized by flow cytometry (BD FACSLyric™ Flow Cytometry System). Centrifuged patients’ plasma (50μl) was thawed and diluted to a final volume of 400μl 1× PBS filtered through a 0.02 μm syringe filter. The custom kit for the evaluation of EVs (BD 626267, 25) was combined according to manufacturer’s suggestion with Trucount™ Tubes (BD Biosciences) for quantification, whereas sizing was determined using the Rosetta Calibration Beads mix and software (Exometry Inc., Amsterdam, Netherlands) which allow as previously described, for correct report of EV diameter based on their refractive index and the FCM configuration, (26). The following membranous antibodies were used for specific staining: CD45 (PerCp-Cy5.5 Mouse Anti-Human CD45;BD564105), CD63 (PE Mouse Anti-Human CD63 BD 556020), and EphA2 (BV421 Mouse Anti-HumanEphA2; BDOptiBuild 748144). The BD custom Kit is based on an APC emitting lipophilic cationic dye that diffuses through the plasma membranes, and Phalloidin FITC as a probe to stain damaged particles. Analysis was performed on dye-positive/phalloidin-negative events (intact EVs) focused on three size groups of approximately: 2μm, 3μm and 5μm. Dye positive events were first evaluated for CD45 to identify hematopoietic-derived EVs. Then CD45-negative (CD45-) or CD45 positive (CD45+) events were gated to define CD63 positive (CD63+) and/or EphA2-positive events (EphA2+). The threshold was set according to manufacturer’s instructions, and the flow rate was medium. A series of quality controls was performed including buffer-only and singles-stained controls which confirmed the absence of detectable events and thus background interference.

### 2.4 Model establishment – problem formulation

In the current study a flexible ML pipeline was implemented to develop data-driven models for PC diagnosis, leveraging supervised learning analysis and histological groups as defined in our dataset. The histological categories for the classification problem under study were the (i) benign epithelial lesions, (ii) exocrine tumors and (iii) endocrine neoplasms. Non-oncological patients were also included in the analysis. To achieve study’s objective, all variables available prior to surgery along with EV-based features extracted from FCM analysis, were included in the models as potential predictors (**Table 1**).

**Table 1:** Description of the features from the FCM data files, including the total number of features utilized in the current analysis for the patients in each use case and the subsequent supervised learning analysis.

Type	Features
Demographics	Sex, Age
Hematological	White blood count (WBC), Red blood count (RBC), Hemoglobin (HGb), Hematocrit (HCT), Mean Corpuscular Volume (MCV), Mean Corpuscular Hemoglobin (MCH), Mean corpuscular hemoglobin concentration (MCHC), Red cell distribution width (RDW), Platelets, Mean Platelet Volume (MPV), Nucleated red blood cells (NRBC), Platelet Distribution Width (PDW)
Biochemical	Glucose, Urea, Creatinine, Potassium, Sodium, Calcium, Magnesium, Serum Glutamic-Oxaloacetic Transaminase (SGOT), Serum Glutamic Pyruvic Transaminase (SGPT), LDH, Alkaline phosphatase, Gamma-Glutamyl Transferase (γGT), Amylase, Total Bilirubin, Direct Bilirubin, Total protein, Albumin, High-sensitivity C-reactive protein (hsCRP), Prothrombin Time, international normalized ratio (INR), Activated partial thromboplastin time (APTT), Hepatitis B surface antigen (HBsAg), Fibrinogen
Histology and Lesion- based	non-oncological, benign, malignant exocrine tumors, endocrine neoplasms
FCM data	CD45 to exclude EVs origin from the hematopoietic system, CD63, member of the tetraspanin family, highly enriched within EV membranes, EPHA2 as a potential biomarker for pancreatic cancer diagnosis as well as combinations of those markers

Two different clinical use cases were defined based on the lesion’s histopathology, each formulated as a 3-class classification problem. Both use cases were applied to FCM data for identifying potential predictors towards PC diagnosis according to the histological groups defined. In the first use case, the classes included patients with benign lesions, exocrine tumors and endocrine neoplasms. In the second use case, the classes comprised of non-oncological patients, patients with exocrine tumors or endocrine neoplasms. The distribution of the classes for the two use cases are presented in **Tables 2**, **3**.

**Table 2:** Distribution of the three classes of the first use case of the FCM analysis.

Target class	Histology type	Number of samples
Class 0	Patients with benign lesions	16
Class 1	Patients with endocrine neoplasms	12
Class 2	Patients with exocrine tumors	72

**Table 3:** Distribution of the three classes of the second use case of the FCM analysis.

Target class	Histology type	Number of samples
Class 0	Non-oncological patients	26
Class 1	Patients with endocrine neoplasms	12
Class 2	Patients with exocrine tumors	72

### 2.5 Machine learning analysis

#### 2.5.1 Data pre-processing and handling of missingness

Specific data preparation techniques were applied to obtain a representation of the raw feature vectors that are better suited for the estimators to be used in the cross-validation scheme proposed in this study. The scikit-learn library (27) for ML in Python (28) was used for building the proposed flexible and comprehensive pipeline. To create a complete dataset for additional analysis, features (mostly patient’s clinical data) having over 30% missing values were removed from the original dataset. For the imputation of the remaining missing values within the dataset, the nonparametric k-NN imputer was applied. The chosen k-value parameter was k=5. The imputation was achieved using the most frequent value within k neighbors for discrete features and mean/mode for continuous variables. A distance function was used to compute how similar two instances were. The default Euclidean distance function provided by the k-NN imputer of the sklearn package was used (27). In addition, the selection of k-value was based on feature similarity and the selection process is known as parameter tuning, which is important for higher accuracy. To apply the k-NN imputer, prior normalization of the data was required to ensure that all features were mapped to the same range as different scales of data can generate biased replacements for the missing values (29, 30). For the normalization part the scikit-learn’s MinMaxScaler was used, which scales features to have values between zero and one (31).

#### 2.5.2 Feature selection

Feature selection was conducted using the scikit-learn meta-transformer, called SelectFromModel (32). SelectFromModel is a meta-transformer that can be applied to any estimator that, after fitting, gives significance to each feature using a callable function or a particular attribute. In the present study, the RF algorithm was employed for assigning weights in each feature to rank them according to their relative importance. In tree-based models, feature importance is typically computed based on how much each feature contributes to reducing impurity (e.g., Gini impurity or entropy) or decreasing the loss function. Specifically, in Random Forest, a feature’s importance score is determined by the total reduction in impurity across all trees in the forest. To identify the most important predictors that contribute to risk prediction of PC diagnosis, the ranking of feature importance scores along with the computed elbow plots were analyzed across all available features of the dataset (**Supplementary Figures 1**, **2** in **Supplementary Material A**). Based on this knowledge, we selected the top 30 features that had the highest importance scores, while the remaining ones provide minimal additional information. Hence, the maximum number of features to be selected by the estimator for subsequent analysis was set to 30. Considering the large number of initial total features derived from both FCM measurements and clinical data, the rationale behind this selection was their association with disease detection. In addition, the Recursive Feature Elimination (RFE) method was also applied as an alternative way to test the feature selection process and hence the performance of the models. The feature selection scheme, in the present pipeline, was applied only on the training phase of the dataset to avoid the rather common issue of data leakage.

#### 2.5.3 Tumor identification with ML algorithms

##### 2.5.3.1 Model training and validation

To avoid the rather common problem of model overfitting in ML models, a 5-fold cross-validation scheme was applied in the training dataset with hold-out data kept for model testing. On this basis, the validation of the model was performed in cases that were not part of the training phase. This approach helps to minimize misclassifications during the training phase while also reducing generalization errors. Furthermore, the trained models were applied to an external subset of data for validation. This dataset was derived from additional patients’ samples (N=38) which were collected and processed in the clinical laboratory following the same methodology with the training dataset, so that any variation due to sample handling would be eliminated. Specifically, samples derived from patients with all the different pathologies were selected exclusively for the validation phase: 11 non-oncological patients, 4 patients with benign lesions, 21 patients with exocrine tumors, 2 patients with endocrine neoplasms. This subset included new, “unseen” samples to assess the model’s performance and predict the class of the new patients (i.e. non-oncological, with benign lesions, exocrine tumors or endocrine neoplasms) which were not used during the training phase.

Hyperparameter tuning was applied to every estimator using the grid search procedure for identifying the best performing model with optimized parameters. This approach involved defining a parameter grid for each algorithm and evaluating various hyperparameter combinations through exhaustive research. A detailed overview of the best hyperparameter values selected for each model is provided in **Table 4**. For LR, the “C” parameter controls the regularization strength, while the “l1” ratio determines the balance between L1 and L2 regularization when using elastic net (ENET). Since the current dataset is approaching a high-dimensional setting, ENET was applied to mitigate the issue of overfitting and multicolinearity. In RF, the “max_depth” parameter limits the tree depth to prevent overfitting while the parameters “min_samples_leaf” and “min_samples_split” control tree growth. The parameter “n_estimators” determines the total number of trees in the model. For SVM, the “gamma” parameter influences data point impact, and the “kernel” parameter defines the transformation function. In XGBoost, “learning_rate” parameter controls step size for updates, “max_depth” manages tree complexity, and the “n_estimators” parameter defines the number of boosting rounds. These hyperparameters were fine-tuned to optimize model performance, balance interpretability and reproducibility while minimizing overfitting, supporting a rigorous approach to multi-class classification problems.

**Table 4:** The optimal parameters based on the Grid Search procedure for each classification model.

ML models	Best parameters	
	First use case	Second use case
LR	{‘C’: 100, ‘l1_ratio’: 0.9’}	{‘C’: 10, ‘l1_ratio’: 0.1}
RF	{‘max_depth’: 10, ‘min_samples_leaf’: 1, ‘min_samples_split’: 2, ‘n_estimators’: 50}	{‘max_depth’: 10, ‘min_samples_leaf’: 1, ‘min_samples_split’: 5, ‘n_estimators’: 100}
SVM	{‘gamma’: ‘scale’, ‘kernel’: ‘poly’}	{‘gamma’: ‘scale’, ‘kernel’: ‘poly’}
XGBOOST	{‘learning_rate’: 0.01, ‘max_depth’: 6, ‘n_estimators’: 200}	{‘learning_rate’: 0.3, ‘max_depth’: 3, ‘n_estimators’: 200}

##### 2.5.3.2 Classification and evaluation of model performance

To build the composite estimator according to the proposed cross-validation scheme on the training dataset, several ML algorithms were applied, namely Logistic Regression (LR), the non-parametric kernel-based model Support Vector Machine (SVM), the ensemble model Random Forest (RF), and Extreme Gradient Boost (XGBoost). Python programming language was used with the scikit-learn ML library (33), for the design and development of a flexible ML-based pipeline for PC diagnosis. Class imbalance handling was addressed based on oversampling functions of the Imbalanced-learn library (34). Over-sampling generates new samples in the classes which are under-represented by random sampling with replacement of the current available samples.

The following metrics were calculated to assess the performance of the classification models: precision, recall (true positive rate), and accuracy. The Receiver Operating Characteristic (ROC) curve was also computed to represent the trade-off between the false negative and false positive rates for every possible cut-off. The weighted One-vs-Rest (OvR) AUC scores while also the pairwise AUC scores were calculated. The OvR AUC score considers each class separately by treating it as a binary classification problem against all other classes, with the final score being an average weighted by class distribution. Hence, OvR offers a broader view of overall class separability. Pairwise AUC scores provide a measure of how well a classifier distinguishes between individual class pairs, offering insights into class separability, ranking consistency, and potential weaknesses in multi-class classification settings (35).

Explainable AI analysis was conducted using SHAP values to determine the key features influencing model predictions (36). A SHAP plot is a graphical representation of SHAP values The features are positioned on the y-axis in a descending order of importance, while the x-axis displays SHAP values, indicating the degree of contribution of each feature towards the model’s prediction. Below zero, the prediction is reduced, while above zero, the prediction is increased for a specific class. Features having a greater impact on model’s prediction (either positive or negative), present a SHAP value away from zero. Each dot displays a unique data point and its color corresponds to lower (e.g. blue color) or higher (e.g. red color) numerical value. Accumulation of higher values on the right side of the graph indicates that the feature positively influences the prediction, suggesting that its increase would benefit the model. Conversely, if higher values are concentrated on the left, an increase in that feature will reduce the prediction. Features displaying a mix of overlapping high and low values (e.g. red and blue dots) suggest non-linear effects, possibly interacting with other features and influencing prediction outcomes.

## 3 Results

### 3.1 First use case

The 3-class classification problem of the first use case included patients with benign lesions, exocrine tumors or endocrine neoplasms. Various ML models were evaluated and their performance in terms of mean and standard deviation of the 5-fold cross validation is presented in **Table 5**. As demonstrated, the RF had the best performance in this case, achieving high classification accuracy. According to the respective confusion matrix (**Supplementary Figure 1** in **Supplementary Material B**) 72 out of 72 samples (100%) were correctly identified for classes 0 and 1 whereas for class 2, 64 out of 72 (89%) were correctly categorized. Regarding the pairwise AUC scores, the model demonstrates consistent and high AUC scores (0.98 across all class comparisons) with minimal variation. This suggests that RF performs equally well across all class pairs, indicating balanced discriminative power among the three classes. These results highlight the model’s robust capability in categorizing the classes with consistent reliability across the multiclass classification task.

**Table 5:** Results of classifier’s evaluation for the first use case of the FCM analysis.

Evaluation Metrics	Classifiers			
	LR (mean+/-SD)	RF (mean+/-SD)	SVM (mean+/-SD)	XGBoost (mean+/-SD)
Balanced Accuracy	0.77 +/- 0.03	0.96 +/- 0.03	0.90 +/- 0.06	0.94 +/- 0.02
Precision Weighted	0.78 +/- 0.04	0.97 +/- 0.02	0.91 +/- 0.06	0.95 +/- 0.02
Recall Weighted	0.77 +/- 0.04	0.96 +/- 0.03	0.90 +/- 0.06	0.94 +/- 0.02
ROC OvR Weighted	0.86 +/- 0.06	0.98 +/- 0.01	0.94 +/- 0.05	0.99 +/- 0.00
Pairwise AUC-ROC (class 0 and class 1)	0.95 +/- 0.00	0.98 +/- 0.01	0.99 +/- 0.01	0.99 +/- 0.01
Pairwise AUC-ROC (class 0 and class 2)	0.83 +/- 0.00	0.98 +/- 0.01	0.94 +/- 0.00	0.98 +/- 0.00
Pairwise AUC-ROC (class 1 and class 2)	0.87 +/- 0.00	0.98 +/- 0.01	0.97 +/- 0.01	0.99 +/- 0.00

**Figure 1:**
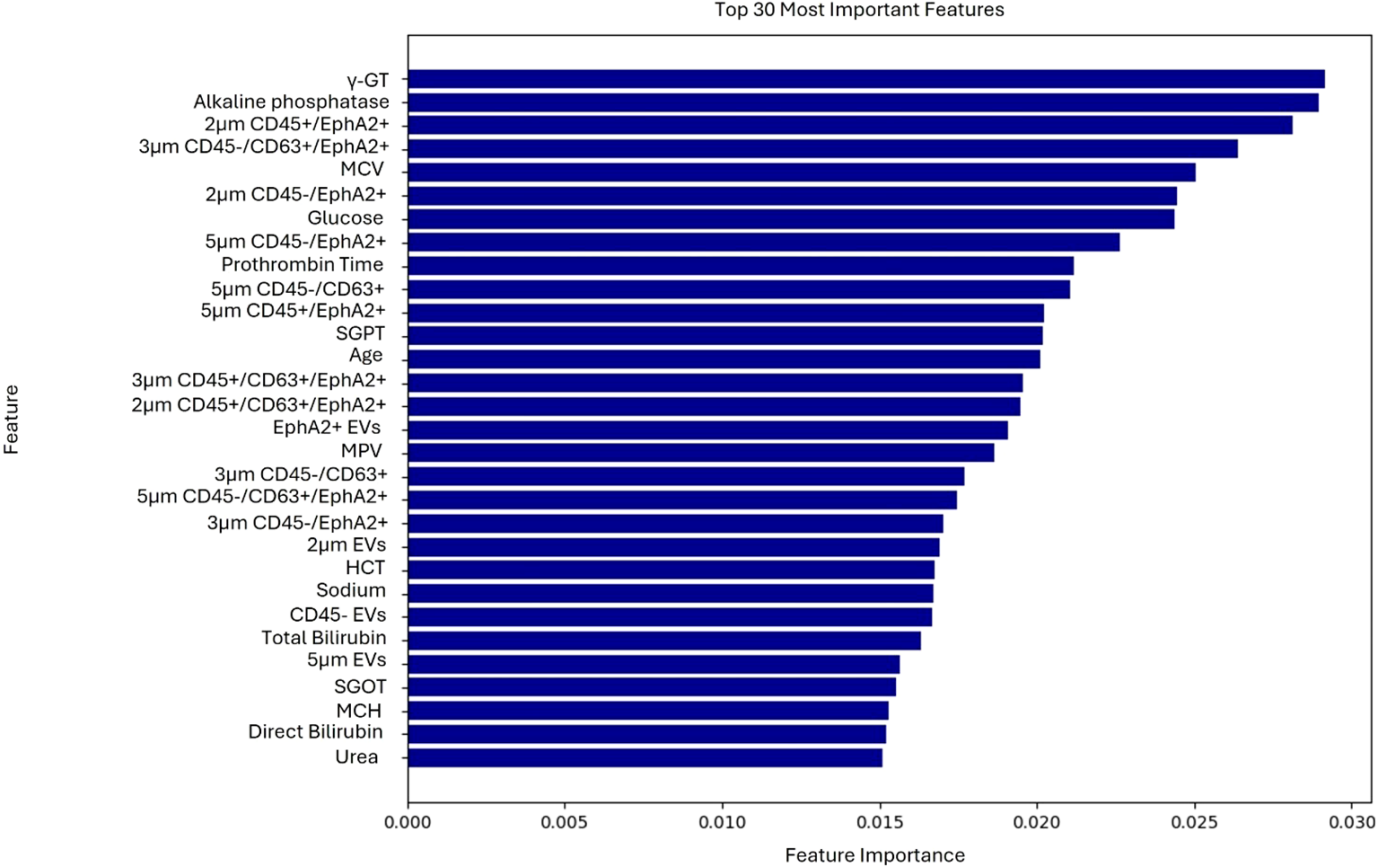
Ranking of the 30 most important features contributing to the model’s predictions of the first use case.

**Figure 2:**
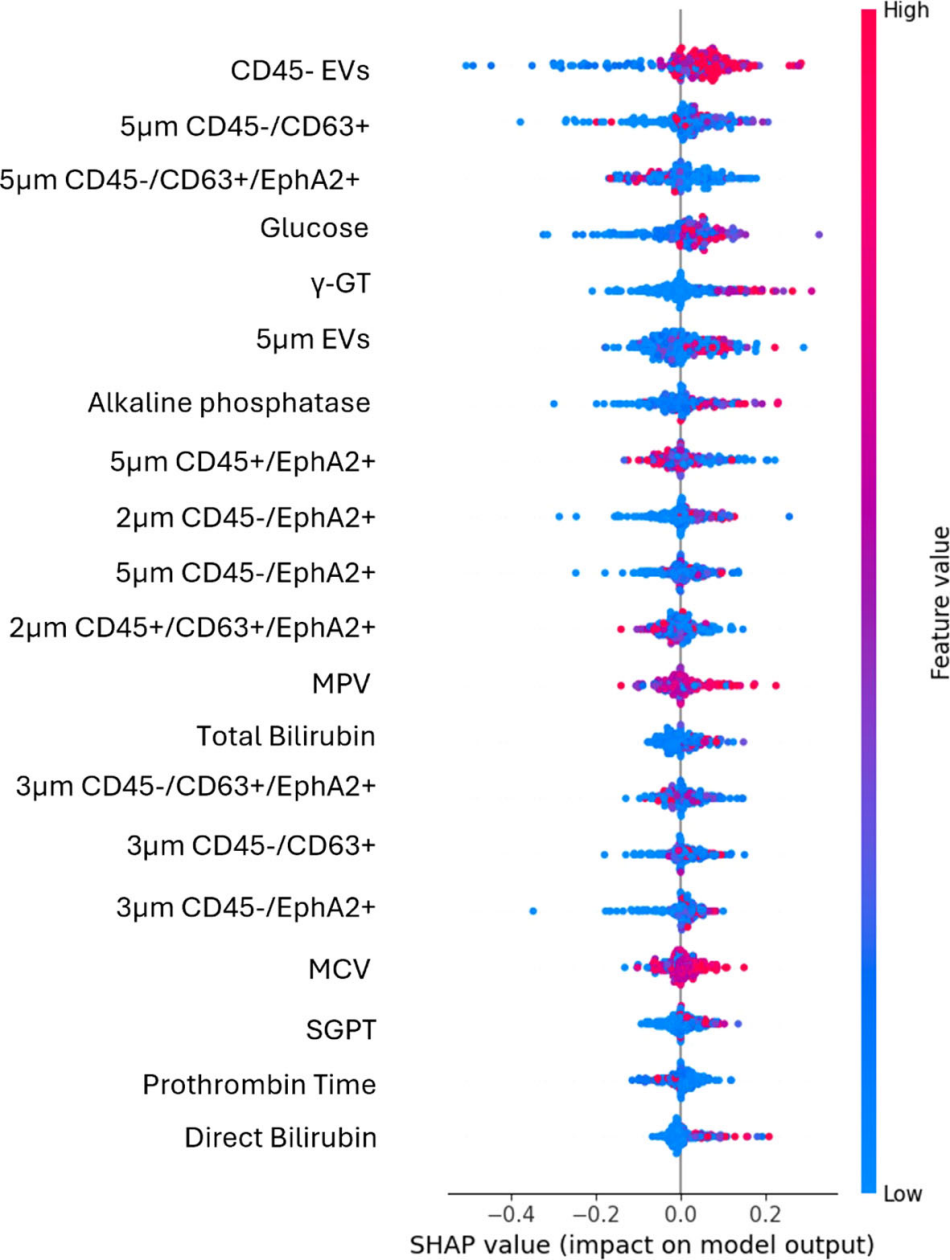
SHAP plot illustrates the contribution of each feature to the predictions of the best performing algorithm of the first use case. High values (red dots) on the right side of the graph suggest that the feature positively influences the prediction, indicating that increasing this feature would benefit the model. High values on the left side suggest that an increase in that feature will lead to a decrease in the prediction.

The model was trained using a combination of EV-based features as well as standard and PC-related biochemical features. The most important features, ranked through the feature selection process, are presented in **Figure 1**. Explainable AI was utilized to interpret the results of the best performing algorithm, which in the present case was RF. Most features selected following the feature selection process were EV-based, and these features predominantly influence model’s predictions as illustrated in the SHAP plot (**Figure 2**).

The most impactful feature was CD45- EVs, as it appeared at the top of the plot with a wide spread of SHAP values, indicating a strong influence on the model’s predictions. The percentages of either the total CD45- EVs population, or its CD63 and EphA2 double positive subpopulation (CD45-/CD63+/EphA2+) were good predictors. At the same time, EVs size alone, specifically EVs of 5μm diameter without any additional biomarker appeared to represent strong prognostic indicators. Additionally, the CD45-/CD63+/EphA2+ subpopulations of different sizes had a significantly impact in model’s output, as EVs in the range of 3μm and 5μm with this molecular profile were key predictive features. The spread of SHAP values for these features showed that their values differentially impact model’s output.

Notably, conventional biochemical markers such as glucose, γ-GT, alkaline phosphatase, total bilirubin, and direct bilirubin were among the top-ranked features. For instance, higher glucose levels contributed more to the model output, indicating a higher probability of classification into the positive class. In contrast, features like prothrombin time and direct bilirubin showed a smaller impact, as their values were centered around zero and ranked lower, signifying minimal contribution compared to higher-ranked features.

### 3.2 Second use case

The second use case of the FCM analysis classified non-oncological patients and patients with exocrine tumors or endocrine neoplasms. The evaluation of the different ML models and their performance in terms of mean and standard deviation of the 5-fold cross validation are presented in **Table 6**. All the evaluation metrics were computed as averages across all classes. The obtained results indicated that XGBoost outperformed the other models, in accuracy and all other evaluation metrics. This algorithm successfully discriminated 67 out of 72 samples (93%) of the class 0, 72 out of 72 (100%) of class 1 and 62 out of 72 (86%) for class 2 (**Supplementary Figure 2** in **Supplementary Material B**). While the OvR ROC AUC score is comparable to the first use case, there is a significant decline in the pairwise AUC ROC score between classes 0 and 2 (0.95 +/-0.00). As a result, XGBoost has difficulty distinguishing between classes 0 and 2 than it does with the other class pairs. In contrast, the highest pairwise AUC ROC score for class 1 and class 2 suggests substantial distinction between the two groups.

**Table 6:** Results of classifier’s evaluation for the second use case of the FCM analysis.

Evaluation Metrics	Classifiers			
	LR (mean+/-SD)	RF (mean+/-SD)	SVM (mean+/-SD)	XGBoost (mean+/-SD)
Balanced Accuracy	0.82 +/- 0.04	0.93 +/- 0.03	0.86 +/- 0.02	0.93 +/- 0.04
Precision Weighted	0.82 +/- 0.04	0.93 +/- 0.03	0.88 +/- 0.02	0.94 +/- 0.03
Recall Weighted	0.82 +/- 0.04	0.93 +/- 0.03	0.86 +/- 0.02	0.93 +/- 0.04
ROC OvR Weighted	0.90 +/- 0.02	0.98 +/- 0.01	0.94 +/- 0.02	0.98 +/- 0.02
Pairwise AUC-ROC (class 0 and class 1)	0.92 +/- 0.00	0.98 +/- 0.01	0.99 +/- 0.01	0.98 +/- 0.00
Pairwise AUC-ROC (class 0 and class 2)	0.87 +/- 0.00	0.97 +/- 0.00	0.88 +/- 0.00	0.95 +/- 0.00
Pairwise AUC-ROC (class 1 and class 2)	0.93 +/- 0.00	0.98 +/- 0.00	0.96 +/- 0.00	0.99 +/- 0.01

**Figure 3:**
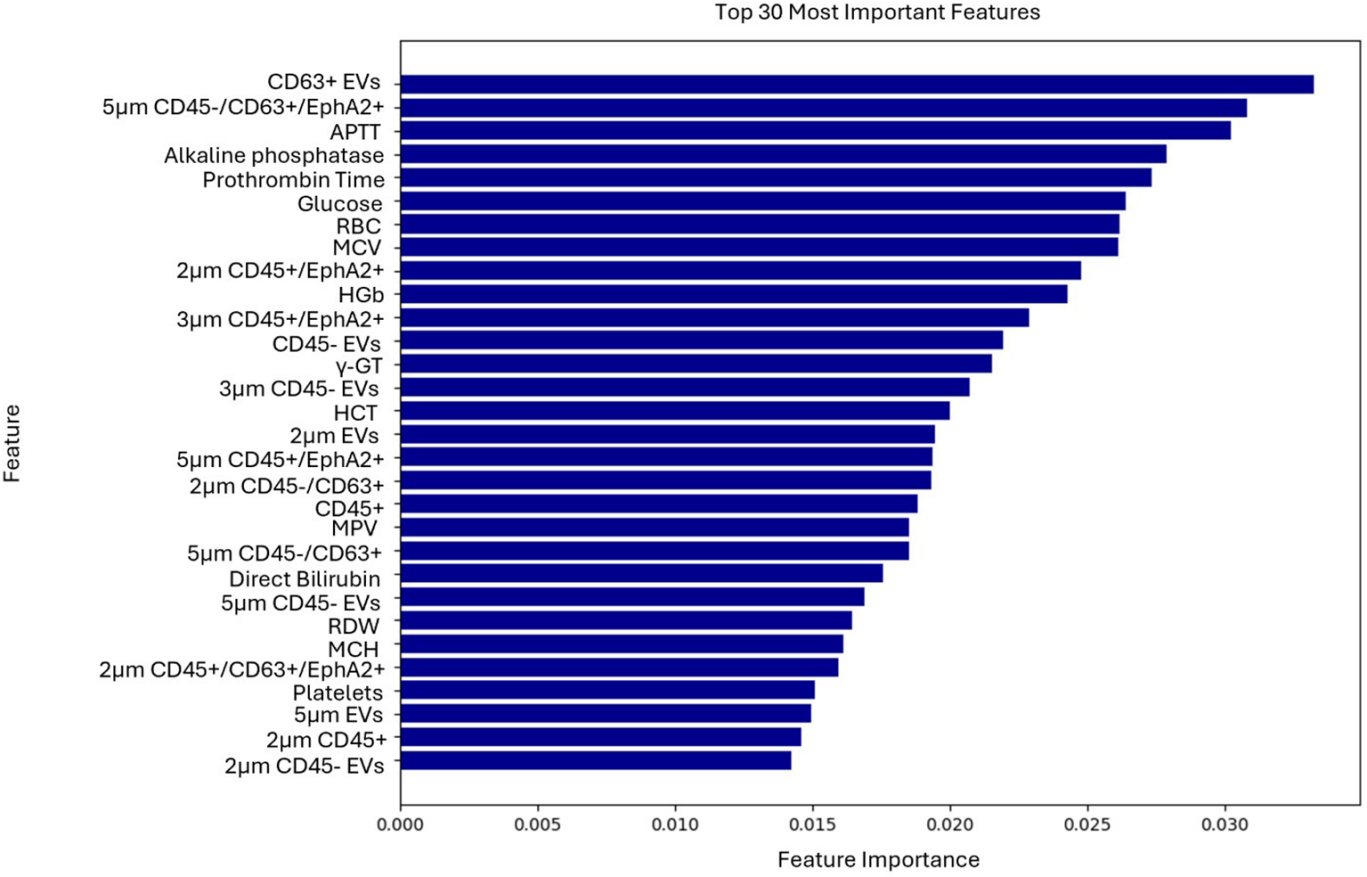
Ranking of the 30 most important features contributing to the model’s predictions of the second use case.

**Figure 4:**
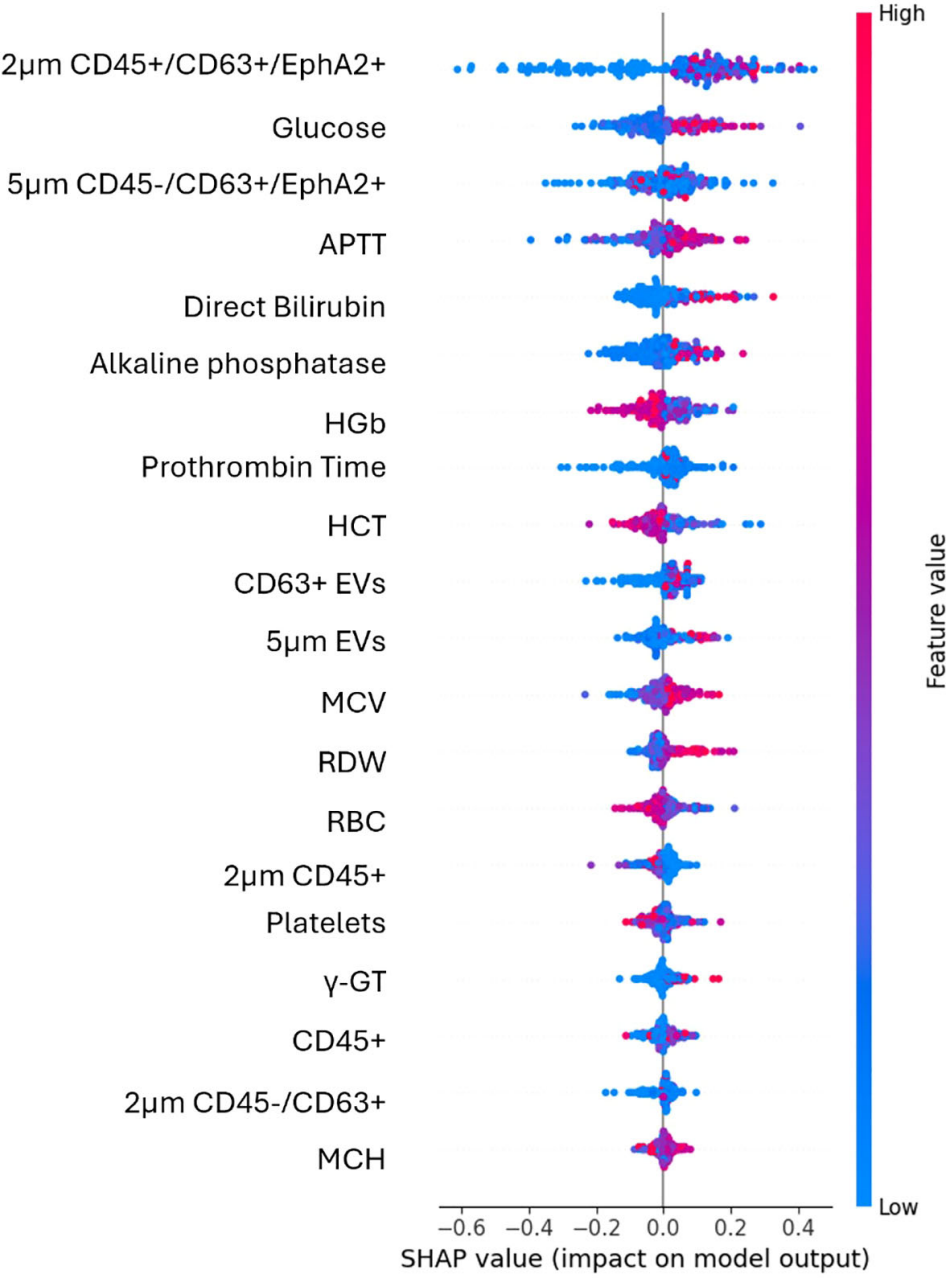
SHAP plot illustrates the contribution of each feature to the predictions of the best performing algorithm of the second use case. High values (red) have a positive SHAP impact when are found on the right side of the plot, suggesting that an increase in feature’s value raises the model’s prediction. Low values (blue) mostly cluster around negative SHAP values and reduce model’s prediction.

Top-ranked features used for the classification of the patients were identified through the feature selection process (**Figure 3**). EV-based features along with some biochemical parameters were among the highly ranked features. The results of the explainable AI analysis conducted with the top-performing algorithm, which in this case was the XGBoost are presented in **Figure 4**.

Among the EV-based features the most impactful for the model’s output were the EVs subpopulation of 2μm in size CD45+/CD63+/EphA2+ and 5μm in size CD45-/CD63+/EphA2+. The distribution of their values was more dispersed across positive and negative SHAP values, indicating a less clear but still significant effect. Significantly impactful features and thus good predictors in this case comprised the percentages of either the total CD45- EVs population, or its CD63+/EphA2+ subpopulation, as well as the 2μm and 5μm sized EVs.

In this case, among biochemical parameters the most impactful was glucose whose higher levels increased the model’s prediction. Features like APTT, direct bilirubin, and alkaline phosphatase showed clear trends with high or low values to influence the model’s positive or negative output. Among the top features, those that present the least impact on the model included MCH, γ-GT and CD45+ EVs.

### 3.3 Validation of model’s performance

The external validation process is critical for assessing the model’s generalization ability to new unseen data. In the present study an external validation set was obtained separately from the dataset used within the training and testing phases to ensure that the evaluation was unbiased and reflective of how the best model would perform on new unseen data. Regarding the external validation process’ assessment metric, we extracted the best-performing model as a “pickle” object by following the guidelines provided by the respective Python package (28). The results of the external validation analysis are summarized in **Table 7**. The scores in **Table 7** refer to the model’s estimated performance on unseen data. It is basically an estimate of accuracy of the model’s performance on new unseen data. In the first case the results indicated that the model performed well at identifying the patients with exocrine lesions but struggled with accurate categorization of patients with benign and endocrine lesions, probably due to class imbalances. In the second case, the model showed robust performance on exocrine and non-oncological samples, while the performance on endocrine samples remained suboptimal. Despite these challenges, the best performing model was deployed without requiring retraining to ensure consistent performance on future data. Furthermore, only the most significant predictors chosen by the best performing model were added to the external validation dataset in order ensure accurate prediction results, considering the significance of real-world data distribution.

**Table 7:** Results of the external validation process.

Analyses	External Validation Scores
First use case	0.77
Second use case	0.80

## 4 Discussion

Accurate differentiation of malignant precursors from their benign counterparts is not always a straightforward decision, while their treatment strategy involving a choice between surveillance and surgery can be challenging (37). From the viewpoint that some of the cysts can potentially be transformed into PC, their surgical resection offers an effective preventive measure for cancer and thereby improving patient survival. However, exposing patients to the morbidity and mortality of unnecessary surgery remains a significant concern for many clinicians and there is a lot of interest in novel methods for classification of patients (37, 38). Liquid biopsy is among the most promising recent advancements in cancer research. Initially, the non-specific predictive potential of ‘exosomes’ count, and size has been proposed to be important and useful for a future general “first-level screening” of cancer risk. Logozzi et al. indicate the very considerable predictive power of exosomes features in cancer and support further research on the relation of structure among non-specific and specific exosome-based biomarkers (39).

The detection of subcellular particles such as EVs with increased purity and yield is a complex function involving multiple factors and pre-analytical variables such as the selection of patient’s plasma over serum and the handling of samples to prevent platelet derived microvesicles or lipoproteins which are also considered confounding factors for EVs. Smaller species of lipoproteins such as high density (HDL, 5–12 nm), low density (LDL, 18–25 nm) which are of similar sizes with small EVs or larger species such as very low-density lipoproteins (VLDL, 30–80 nm) or chylomicrons (CM, 75–1200 nm, <0.930 g/cm^3^) can be found in high densities in patients’ plasma (9, 40). To address this as well as reduce any donor’s related variability, standardized sample collection procedures, including patient fasting that has been described to significantly reduce the amount of larger lipoprotein species, were implemented (40). Blood was collected from participants just before entering the operating room following a similar fasting period. Furthermore, as confounding factors such as age and gender should always be considered, in the present cohort attention was paid so that participants were of similar age (41). Storage was also standardized to ensure experimental reproducibility. Moreover, the presence of EVs in a small number of patient samples has already been validated using Western blot, which is commonly employed as part of a multi-method approach to detect EV-associated proteins (42).

The analytical approach implemented to detect and characterize PC-derived EVs in the present study provided valuable information to the ML model, qualifying it to discriminate with high accuracy non-oncological patients, patients with benign lesions, or exocrine tumors or endocrine neoplasms. FCM variables provided estimates of particle size by light scatter, concentration, plus a molecular phenotyping of its targets via fluorescence. Despite its limitations and challenges such as the antibody-dependent cost or finding a universal standardization strategy, FCM is widely recognized as a technology platform and the most viable technique for analyzing and adequately describing EVs. Numerous data based on FCM were collected to assist EVs characterization and their contribution to liquid biopsy’s potential for cancer early detection (10, 43–45).

Previous studies investigating the diagnosis of PC through the detection of EVs employed different approaches for EV isolation and primarily relied on statistical analysis rather than ML of EV-based biomarkers, as summarized in **Table 8**. Yoshioka et al., detected through ultracentrifugation EV-associated proteins as potential biomarkers in a small dataset of PC patients, chronic pancreatitis (CP) patients and healthy controls. Their findings highlighted EV-associated GPRC5C and EPS8 as highly accurate biomarkers, with AUC values of 0.922 and 0.946 (21). Similarly, Odaka et al. explored the diagnostic potential of EVs by analyzing CD63+ EVs and platelet-derived EVs (CD41+ and CD61+) by means of sandwich enzyme-linked immunosorbent assay (ELISA). This approach yielded an AUC of 0.846 in distinguishing PC patients from healthy controls (22). Moreover, Wei et al. suggested the combination of EphA2 expression in exosomes with carbohydrate antigens CA19.9 and CA242 in patients’ serum, as a valuable biomarker (24). Additionally, exosomal surface proteins GPC1, CD82 along with CA19.9 were tested in 76 participants as potential biomarkers from Xiao et al., achieving a high AUC of 0.942 (23).

**Table 8:** Previous studies with EV-based biomarkers.

First Author, Year	Biomarkers	Technique	Dataset
Yoshioka et al., 2022 (21)	EV-associated proteins	Statistical analysis	PC: 54, CP: 22, HC: 32
Odaka et al., 2022 (22)	EVs (CD63+-EVs) or platelet-derived EVs (CD41+- and CD61+-EVs)	Statistical analysis	PC: 39, HC: 39
Wei et al., 2020 (24)	Expression of serum Ephrin type-A receptor 2 in exosomes (Exo-EphA2), CA 19.9 and CA 242	Statistical analysis	PC: 204, PBD:75, HC: 74
Xiao et al., 2020 (23)	Exosomal surface protein glypican-1 (GPC1), exosomal cluster of differentiation 82 (CD82), and serum carbohydrate antigen 19.9 (CA19.9)	Statistical analysis	76 participants

CP, Chronic pancreatitis; HC, healthy controls; PBD, pancreatic benign disease.

In the current, new to the field, ML approach, two alternative methods i.e., (i) SelectFromModel and (ii) RFE—were used in the feature selection process to determine which features were most crucial for differentiating the different patient groups in each scenario. The SelectFromModel meta-estimator was chosen as the main feature selection technique since it produced the most representative results out of all. The accurate performance of this technique can be attributed to its reliance on model-based importance, which often provides a more direct measure of feature relevance. To address the significant class imbalance in the dataset, both oversampling and undersampling techniques were tested. However, due to the dataset’s limited size and the extreme imbalance between the classes, only oversampling yielded favorable results. In terms of ML analysis all the algorithms that were tested demonstrated good performance. The two best performing algorithms, depending on the use case were XGBoost and the RF. While XGBoost generally outperforms RF in accuracy, it can be more sensitive to overfitting, especially if hyperparameters are not carefully tuned (46). Conversely, RF might provide a faster and more straightforward solution in cases of smaller datasets or simpler tasks as in this case. The external validation process, which is critical for assessing the model’s generalizability to new, unseen real-world data, produced high scores, corroborating the robust accuracy achieved during model training. However, the model demonstrated difficulty in discriminating between patients with exocrine tumors, endocrine neoplasms and some benign lesions, likely due to the small number of patients with endocrine and benign histopathology compared to patients with exocrine tumors as a result of disease incidence.

The biological interpretation of the output indicates that the detection of larger vesicles (FCM; ~2μm – ~3μm – ~5μm) constitute an efficient and accurate way to reflect the medical status of PC patients, scoring over 0.90 in accuracy of the RF and XGBoost algorithms (i.e., 0.96 +/- 0.03 accuracy in the first use case and 0.93 +/- 0.04 in the second use case) as well as the other evaluation metrics (**Tables 5**, **6**), further highlighting the importance of circulating EVs. Special attention should be given to the selected triplet of markers CD45, CD63, EphA2 that proved to add adequate information about the molecular profile of EVs contributing to the distinction between PC patients and patients with benign lesions or non-oncological patients. This approach and its output stand out compared to other studies which test combinations of more than three protein biomarkers on EVs without significantly enhancing precision or accuracy (47). EVs subpopulations such as CD63+ EphA+ EVs were previously described to be significantly over expressed in PC cell-lines compared to normal pancreatic cell line as well as in PC patient serum, with high diagnostic potential when combined with the traditional CA19.9 (AUC 0.958, P ¼ 0.0007) (48). Moving one step further, in the current study the high impact of CD45- EVs values and its subpopulations was mostly observed in the first use case that assist the classification of patients with benign lesions, exocrine tumors or endocrine neoplasms. Conversely, in the second use case, where non-oncological patients were included CD45- EVs subpopulations presented with a smaller impact.

Finally, parameters derived from the hematological and biochemical analysis of patients’ serum such as Prothrombin time, Red blood cell (RBC), Red cell distribution width (RDW), Mean Corpuscular Volume (MCV), Mean Platelet Volume (MPV), platelets, and activated partial thromboplastin time (APTT) were identified as important features in the model, which coincides with the nature of neoplastic process and the fact that PC is characterized by the dissemination of tumor-derived microvesicles, high tumoral expression of tissue factor and activation of leukocytes which all promote hypercoagulability and increased platelet activation (49, 50). Moreover, increased levels of serum bilirubin, alkaline phosphatase, and γ glutamyl transferase (γGT) are often observed as obstructive jaundice is a usual symptom of PC (50). These frequently nonspecific features were recognized as playing an important role in the second use case surpassing most of the EVs based features in significance, with impactful contribution to the model’s ability to discriminate among non-oncological patients and patients with exocrine tumors or endocrine neoplasms. Still, 5μm in size CD45-/CD63+/EphA2+ EVs population remained among the top three predictors.

## 5 Conclusion

In conclusion, this study further supports the great potential of physical properties and molecular profile of EVs to substantially inform and guide clinical decisions with the assistance of a ML algorithm, as an innovative, rapid, and efficient method. The ML algorithm with its capacity to discriminate among patients with different pathologies, either exocrine tumors or endocrine neoplasms, non-oncological patients or patients with benign cysts could represent a valuable tool for clinicians and their patients to improve PC detection at a treatable stage.

Clinical application of non-invasive and effective biomarkers for early detection of PC remains an open issue. Despite the existing difficulties in the preanalytical standardization of the EVs related analysis the presented findings support liquid biopsy’s potential for early detection and monitoring of cancer offering a way to personalize treatments in a fast-evolving landscape. Increasing the patient’s cohort and repeating the measurements during patients follow up should further substantiate the results and the accuracy of the algorithm in each use case. In parallel, given the absence of instruments of higher resolution and low limits of detection that would effectively discriminate EVs of smaller size (e.g. exosomes or microvesicles), the fact that large EVs provide highly valuable information for PC early diagnosis, allows a more affordable algorithm in everyday clinical practice and urge for the thorough biological characterization of these large EVs, as it could further contribute to our understanding of EVs role in PC development and progression.

## Data Availability

The raw data supporting the conclusions of this article will be made available by the authors, without undue reservation.
