# Supplementary material for: A machine-learning approach for pancreatic neoplasia classification based on plasma extracellular vesicles
Source: Front Oncol. 2025 Apr 25;15:1540195. doi: 10.3389/fonc.2025.1540195 (PMC12061713; doi:10.3389/fonc.2025.1540195)
Supplement: Supplementary file 1 [file DataSheet1.pdf]

Supplementary Material

1 Supplementary Data

Supplementary Material should be uploaded separately on submission. Please include any supplementary data, figures and/or tables.

Supplementary material is not typeset so please ensure that all information is clearly presented, the appropriate caption is included in the file and not in the manuscript, and that the style conforms to the rest of the article.

2 Supplementary Figures and Tables

For more information on Supplementary Material and for details on the different file types accepted, please see here.

2.1 Supplementary Figures

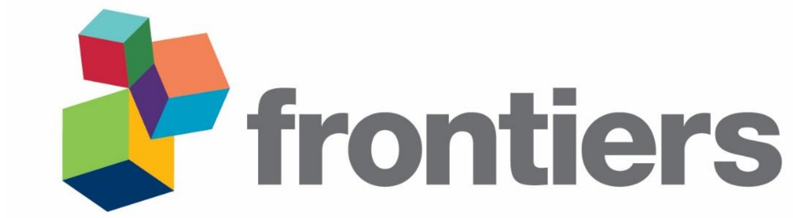

Supplementary Figure 1. The figure legends are required to have the same font as the main text, 12 point normal Times New Roman, single spaced. Please use a single paragraph for each legend and prepare the figures keeping in mind the PDF layout.

Supplementary Material A – Elbow Plots

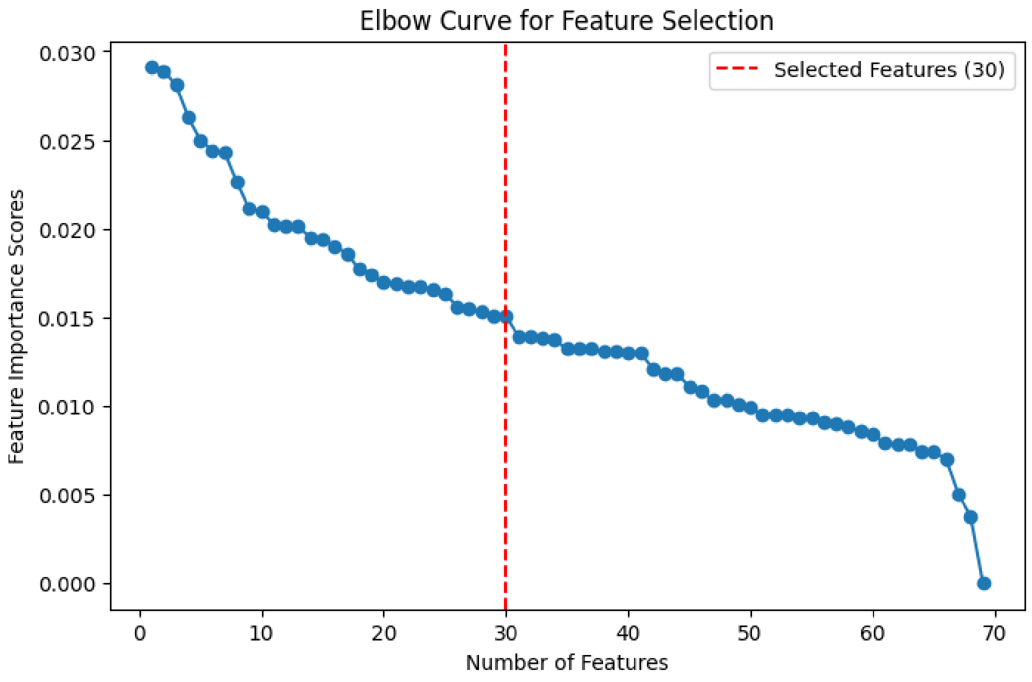

Supplementary Figure 1. Elbow plot for the first use case.

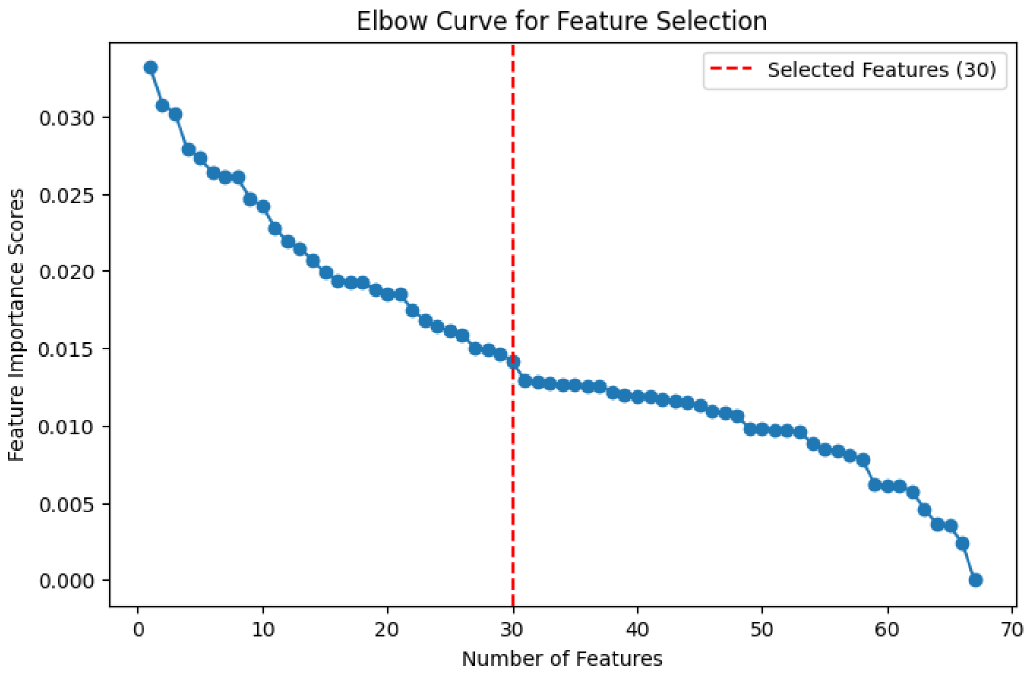

Supplementary Figure 2. Elbow plot for the second use case.

Supplementary Material B - Confusion Matrices

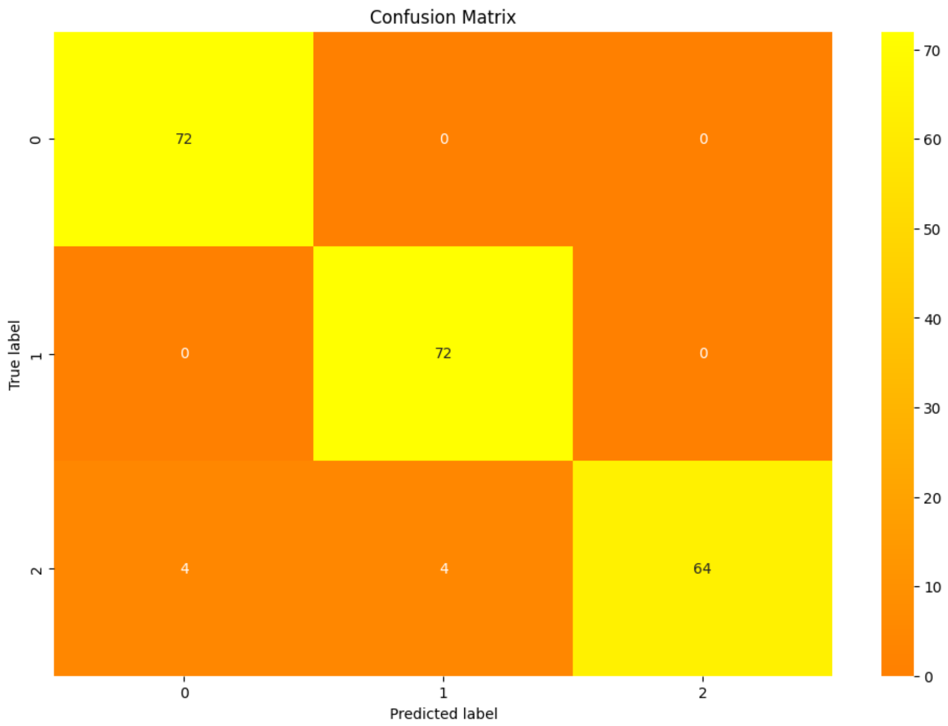

Supplementary Figure 1. Confusion matrix of the best performing algorithm of the FCM analysis of the first use case.

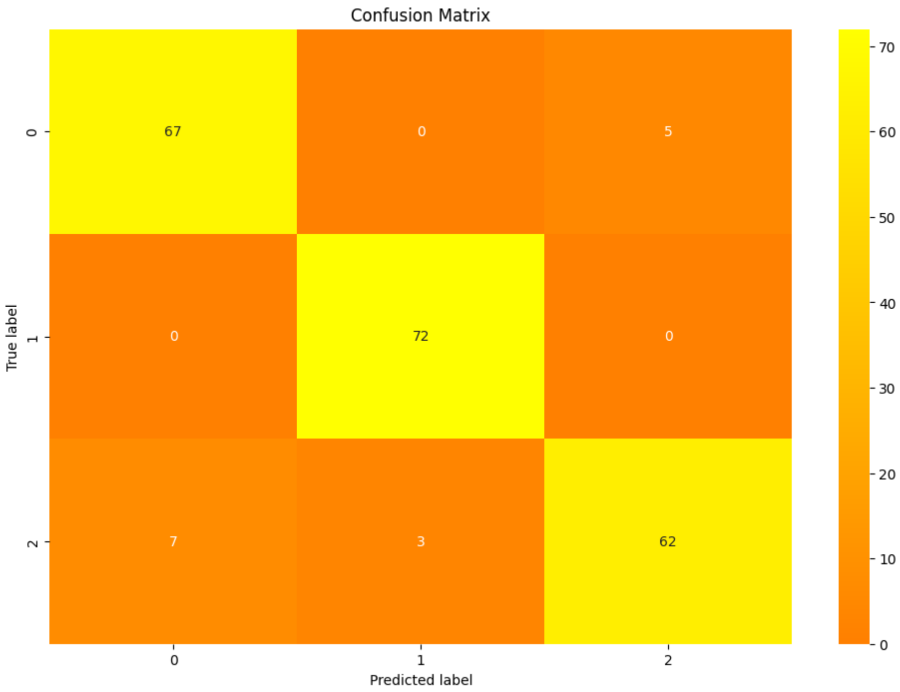

Supplementary Figure 2. Confusion matrix of the best performing algorithm of the FCM analysis of the second use case.
